# Neuralgia Caused by Exostosis

**Published:** 1884-11

**Authors:** 


### Neuralgia Caused by Exostosis.

A lady suffered from intense neuralgia in the region of the second and third branches of the trigemini; especially during mastication, the pain in the right superior maxillary bone was so violent as to force the lady to chew only on the left side of her mouth. These neuralgic attacks, which were really very severe, had existed for several years. As the patient had besides been several times the victim of inflammation of the right external meatus, Dr. Moos, who attended her, and who reports the case in the *Berl. Kl. Woch.* 8, 1884, inquired further into the history of the neuralgia, and found that it always seemed to start from a definite point in the same meatus. Ocular inspection revealed the presence of a small exostosis of the size of a split pea in the meatus. There were two other exostoses in this passage, both anterior to the one first-mentioned, but Moos soon discovered that the posterior one was alone sensitive. This exostosis was, therefore, removed with the straight chisel, and the pains at once ceased, and have since, nearly ten months, not returned. As a remarkable fact may be mentioned, in conclusion, that while the pain specially affected the second branch of the nerve, it started in the third, upon which the pressure was exerted by the exostosis.

It is not in all neuralgias that we can find a starting point. But we should always look for one; and if determined, it will generally be observed that some new growth exerts pressure on the nerve. This new growth may either be a tumor or the product of inflammation, as thickening and swelling of adjoining tissues, or accumulation of matter, as is frequently noticed in cases of a carious tooth, where a small abscess forms at the root of the tooth and causes the neuralgia by tension, *i. e.*, pressure upon the nerve.

